# Characteristics of the disastrous debris flow of Chediguan gully in Yinxing town, Sichuan Province, on August 20, 2019

**DOI:** 10.1038/s41598-021-03125-x

**Published:** 2021-12-08

**Authors:** Ning Li, Chuan Tang, Xianzheng Zhang, Ming Chang, Zhile Shu, Xianghang Bu

**Affiliations:** 1 School of Emergency Science, Xihua University, No.9999 Hongguang Rd, Chengdu, 610039 Sichuan China; 2 State Key Laboratory of Geohazard Prevention and Geoenvironment Protection, Chengdu University of Technology, Chengdu, 61005 China; 3 State Grid Sichuan Electric Power Research Institute, Chengdu, 610072 Sichuan China

**Keywords:** Environmental sciences, Natural hazards

## Abstract

On August 20, 2019, at 2 a.m., a disastrous debris flow occurred in Chediguan gully in Yinxing town, China. The debris flow destroyed the drainage groove and the bridge at the exit of the gully. In addition, the debris flow temporarily blocked the Minjiang River during the flood peak, flooding the Taipingyi hydropower station 200 m upstream and leaving two plant workers missing. To further understand the activity of the debris flow after the Wenchuan earthquake, the characteristics of this debris flow event were studied. Eleven years after the Wenchuan earthquake, a disastrous debris flow still occurred in the Chediguan catchment, causing more severe losses than those of earlier debris flows. In this paper, the formation mechanism and dynamic characteristics of this debris flow event are analysed based on a drone survey, high-definition remote sensing interpretations and other means. The catastrophic debris flow event indicates that debris flows in the Wenchuan earthquake area are still active. A large amount of dredging work in the main gully could effectively reduce the debris flow risk in the gully. In addition, it is also important to repair or rebuild damaged mitigation measures and to establish a real-time monitoring and early warning system for the high-risk gully.

## Introduction

At 2:28 pm on May 12th, 2008, an MS8 earthquake struck Wenchuan County in Sichuan Province (“5.12” earthquake), China^1^. The earthquake induced more than 56,000 landslides in a mountainous area of 41,750 km^2^. Most of the landslides were distributed in hills and gorges, and the landslides converted into frequent debris flows under heavy rainfall after the earthquake^2, 3^. During the 11 years that have passed since the Wenchuan earthquake, the long-term debris flow activities occurring after the earthquake have caused great threats to local residents. Among them, the most serious debris flows occurred on August 14, 2010, in Longchi town and Yingxiu town and on July 10, 2013, in Wenchuan^4, 5^. Some scholars have pointed out that strong debris flow activities after earthquakes will last 10 ~ 15 years or even 30 years^6, 7^. This concept is well supported by the mass debris flow that occurred in Wenchuan County on August 20, 2019.

From 0:00 to 7:00 on August 20, 2019, the cumulative rainfall in Wenchuan County reached a maximum of 65 mm, resulting in a number of debris flows and flash floods and affecting more than 10 townships in the county. Among them, the disasters in Miansi town, Sanjiang town, and Yinxing town were the most serious. According to the information released by the Wenchuan County People's Government, the “8.20” disaster damaged many roads, pieces of communication equipment, farmlands and houses. The local government transferred a total of 48,000 people and resettled 2,505 people. As of September 5, 2019, the debris flows and flash floods caused 16 deaths and 25 missing people, resulting in direct economic losses of approximately 3.408 billion yuan^8^. According to the survey, the losses were mostly caused by flash floods. The debris flow events mainly caused damage to the bridges and infrastructure along the Minjiang River, hindering the transportation, power supply and communication in the disaster area and increasing the difficulty of rescue but not causing casualties.

**Figure 1:**
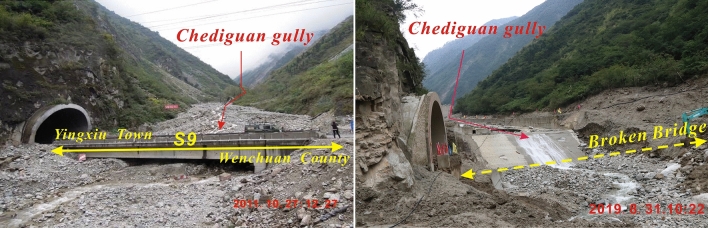
Gully mouth photos of Chediguan gully after the debris flow in 2011 and 2019.

On August 20, 2019, at 2 a.m., debris flow occurred in Chediguan gully of Yinxing Town, with a volume of 63.8 × 10^4^ m^3^. The debris flow poured out along drainage channels and destroyed the drainage groove located at the ditch as well as the G213 Taiping Middle Bridge. According to the photos taken by the UAV, the debris flow fan was 300 m long, 260 m wide, and the average depth is 8 m. The volume and area of the deposition area were 63.8 × 10^4^ m^3^ and 7.9 × 10^4^ m^2^, respectively (Fig. 1). The debris flow front blocked Minjiang River and caused the water level to rise, flooding the active dam area and leaving two people missing.

As Chediguan gully is located along on the only routes of the Du-Wen highway and 213 national road, Chediguan debris flow disasters always threaten the roads and passing vehicles. After the completion of mitigation measures in 2011, the debris flow activity in Chediguan gully was significantly reduced, and the debris flow risk gradually became ignored. The debris flow event in 2019 brought the Chediguan gully back into our view and left us with many questions. The most important question involves the cause of the disaster. What we need to know is why the Chediguan gully, under the defence of mitigation measures, can still break out in such a large-scale debris flow 10 years after the earthquake and cause such serious damage. Based on this concern, we investigated the debris flow disaster that occurred on August 20, 2019, and analysed the formation mechanism and dynamic characteristics of this debris flow event by means of a drone survey, high-definition remote sensing interpretation and other means. This paper can provide a reliable scientific basis for the management of the Chediguan catchment and enrich the understanding of post-earthquake debris flows.

## Study area

**Figure 2:**
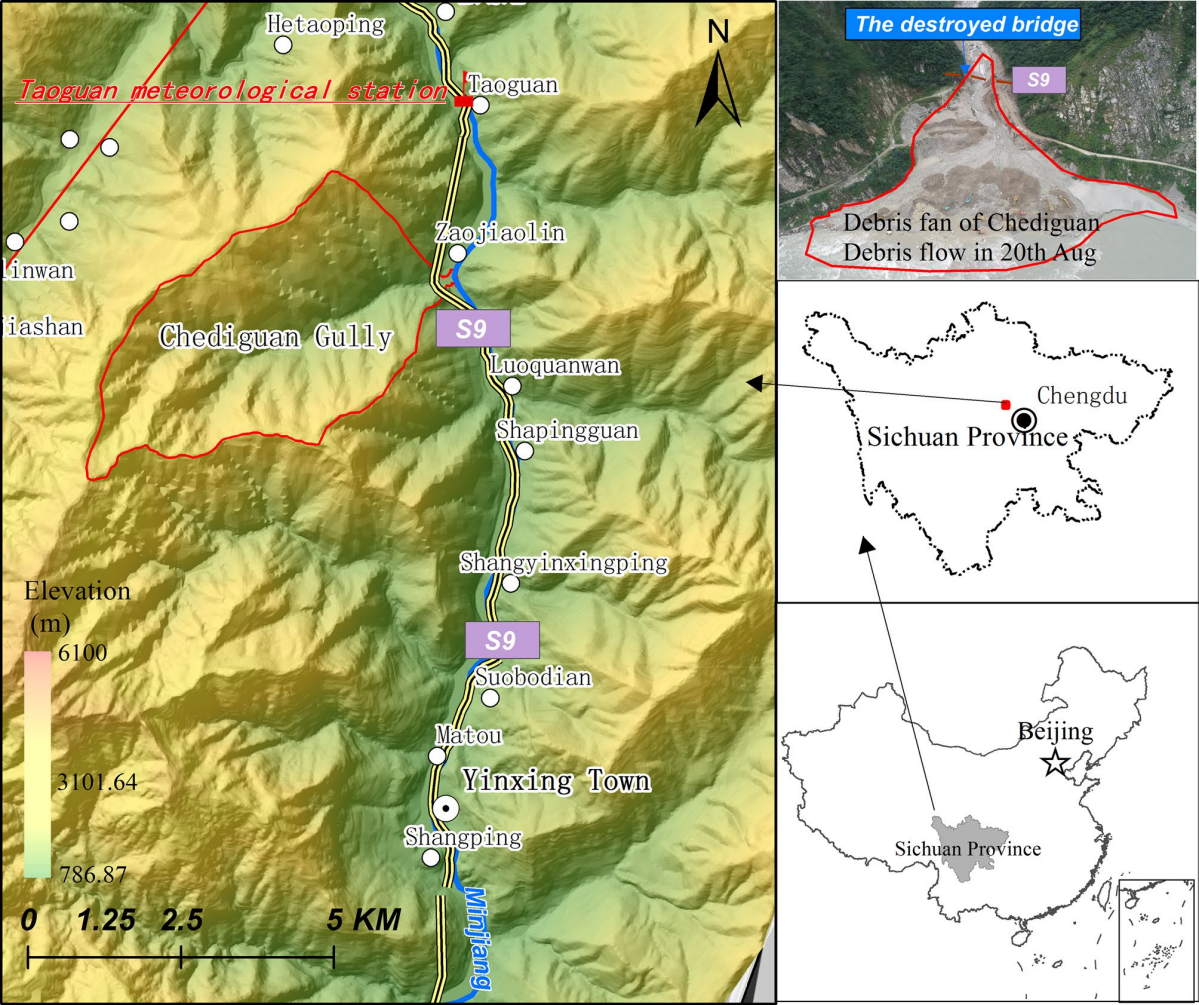
Location map of the study area. Maps constructed using ArcGIS 10.2 (https://desktop.arcgis.com/en/).

Chediguan gully is located in Yinxing town, Wenchuan County, Sichuan Province, China (Fig. 2). The coordinates of its gully mouth are 103°29′2″ E and 31°13′23.4″ N. The flow direction of the gully is from west to east, and the gully covers an area of 16.49 km^2^. The main ditch is approximately 6.2 km long with an average longitudinal slope of 302‰. Maximum altitude 2940 m, minimum altitude 1065.5 m, and the height difference 1574.5 m. The study area is approximately 18 km away from Yingxiu town and approximately 35 km away from Wenchuan County. The Yingxiu-Wenchuan Expressway runs through the ditch along the tunnel and provides convenient transportation. The geographical location of the gully is shown in Fig. 2.

**Figure 3:**
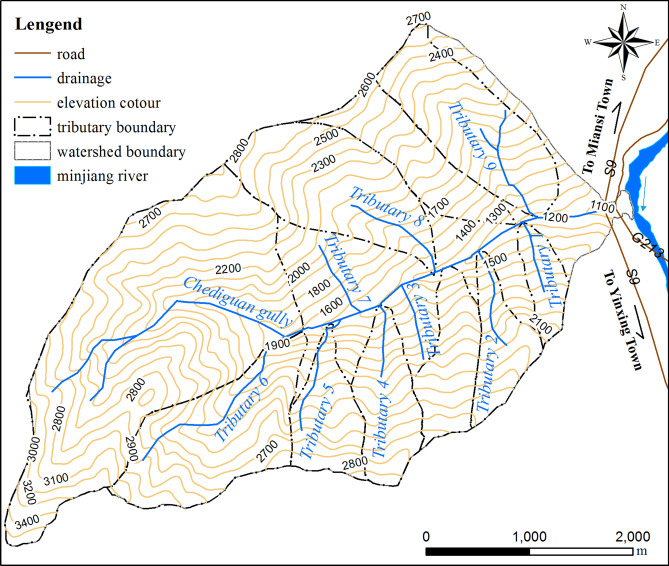
Drainage system and topography map of the Chediguan gully. Maps constructed using ArcGIS 10.2 (https://desktop.arcgis.com/en/).

Chediguan gully belongs to middle and high mountainous areas, with steep overall terrain and free face development in the catchment. The drainage system in Chediguan gully is developed, and there are a total of 9 tributaries along the main channel. All the tributaries are steep, and their lengths and watershed areas are quite different from each other. Compared with the tributaries on the left bank, the tributaries on the right bank are shorter, their areas are smaller, and their slopes are steeper. The developed drainage system can not only effectively collect rainfall but can also promote the migration of source material in the basin to the main channel, thus promoting the formation of debris flows. The drainage system and a topographic map of Chediguan gully are shown in Fig. 3.

The catchment is mainly formed by steep slopes, which are beneficial for accumulating rainfall and causing landslides. Among the whole catchment, gently sloping lands (< 25°) accounting for 11.52% of the total area, which distributed in the downstream area. Steep lands (25°–35°) accounting for 8.79% of the total area, and acutely steep lands (≥ 35°) accounting for 79.69% of the total area. The main channel slope is steeper at and above the intersection with the 6th tributary (Feixianyan gully) but gradually slows below this confluence.

The geological environment of the study area is complicated. It is located in the middle of the Longmenshan-Huaxia tectonic belt with a NE direction of 40° ~ 50°, approximately 14 km away from the Yingxiu fault on the southeast side and approximately 15 km away from the Mao-Wen fault on the northwest side. The lithology of the strata that are exposed along the main channel mainly include biotite granite, Quaternary alluvial-diluvial deposits and seismic deposits resulting from fragmented stones, soils, and debris flow sediments. Affected by the Maowen fault, the biotite granite is severely broken, the thickness of the weathered layer is large, and multiple structural tectonic fissures are developed in the rock stratum, easily forming collapses and landslides, and then into debris flow In the middle and lower parts of the basin, secondary faults are distributed along the ditch that belong to the secondary fault structure of the Longmenshan central fault, and the two sides of the main channel have critically collapsed.

There are frequent earthquakes in the study area. According to the current records, there have been 8 major earthquakes and many strong earthquakes of magnitude 5 or higher. For example, the Diexi MS7.3 earthquake occurred on August 25, 1993, and the Wenchuan MS8.0 earthquake occurred on May 12, 2008. Based on “the ground motion parameter zoning map of the Wenchuan earthquake” (GB18306-2001), the study area belongs to a high-intensity seismic region (VIII degrees) with a peak ground acceleration of 0.2 g and a characteristic period of the seismic response spectrum of 0.4 s. Frequent earthquakes result in loose rock and soil structures in the ditch and abundant reserves of original provenance. According to a survey, the total amount of loose source materials in 2011 reached 742.679 × 10^4^ m^3^, and the reserves were 194.525 × 10^4^ m^3^ in volume^9^. The abundant source material provides favorable conditions for debris flows.

The study area is located in a subtropical humid climate zone and is a concentrated area of heavy rainfall in the Minjiang River Basin. The average annual rainfall in this area is 1253.1 mm, the maximum annual rainfall is 1688 mm, and the minimum annual rainfall is 836.7 mm. The maximum continuous rainfall for 4 months (June–September) is 853.2 mm, comprising 68.2% of the annual rainfall. The rainfall in the survey area is abundant and concentrated and can meet the hydrodynamic conditions required to stimulate debris flows.

Debris flows broke out several times in Chediguan gully after the “5.12” earthquake (Fig. 1 and Table 1). According to the investigation and interview, this gully belongs to an old debris flow gully, and there have been four mudslides in recorded history (Table 1). One mudslide occurred in the year 1952^10^, but its scale and the damage caused by it have remained unascertained for a long time. The second mudslide occurred on the evening of August 13, 2010^11^. When the accumulated rainfall reached 102.8 mm and the hourly rain intensity reached 14.2 mm/h, debris flows broke out in the tributaries on both sides of the gully, but no debris flow occurred in the main channel. The third mudslide occurred on July 3, 2011. Affected by heavy rain, a debris flow comprising approximately 1.5 × 10^4^ m^3^ of material was washed down from upstream and accumulated in the downstream main channel, destroying the drainage canal on the upper side of the Ying-Wen Highway and some mechanical equipment. The fourth mudslide occurred on July 20, 2011. Heavy rainfall caused a large-scale debris flow in the early morning. The total amount of rushing material reached 10 × 10^4^ m^3^, causing the lateral displacement of the G213 bridge offset by 12 cm and interrupting traffic for 2 d. At the same time, more than 1/3 of the Minjiang River was blocked by debris.

**Table 1:** Typical debris flow events of Chediguan Gully.

Time	Barrier dam parameters (m)			Magnitude (× 10^4^ m^3^)	Rainfall (mm)		Impact	Data source
	Max length	Max width	Average depth					
1952	–	–	–	–	–	–	No casualties or economic damage were caused	^9, 10^
2010-8-13	–	–	–	–	19.5 (1 h)	55.3 (72 h)	Debris flow broke out in the tributary	^11^
2011-7-3	–	–	–	1.5	14.2 (1 h)	102.8 (72 h)	The debris flow drainage canal under construction and some mechanical equipment were destroyed	^10, 12^
2011-7-20	100	348	15	52.9	40 (1 h)	300 (72 h)	G213 bridge body offset 12 cm, the tunnel under construction in the ditch partial collapse, the debris flow body temporarily blocked the Minjiang River	^10, 12^
2019-8-20	300	260	8	63.8	17.8 (1 h)	43.2 (24 h)	Two people are missing. Two Bridges, one house and about 650 m country road were destroyed	Field investigation and the Wenchuan County Meteorological Bureau

## Data and methodology

### Field investigation

**Figure 4:**
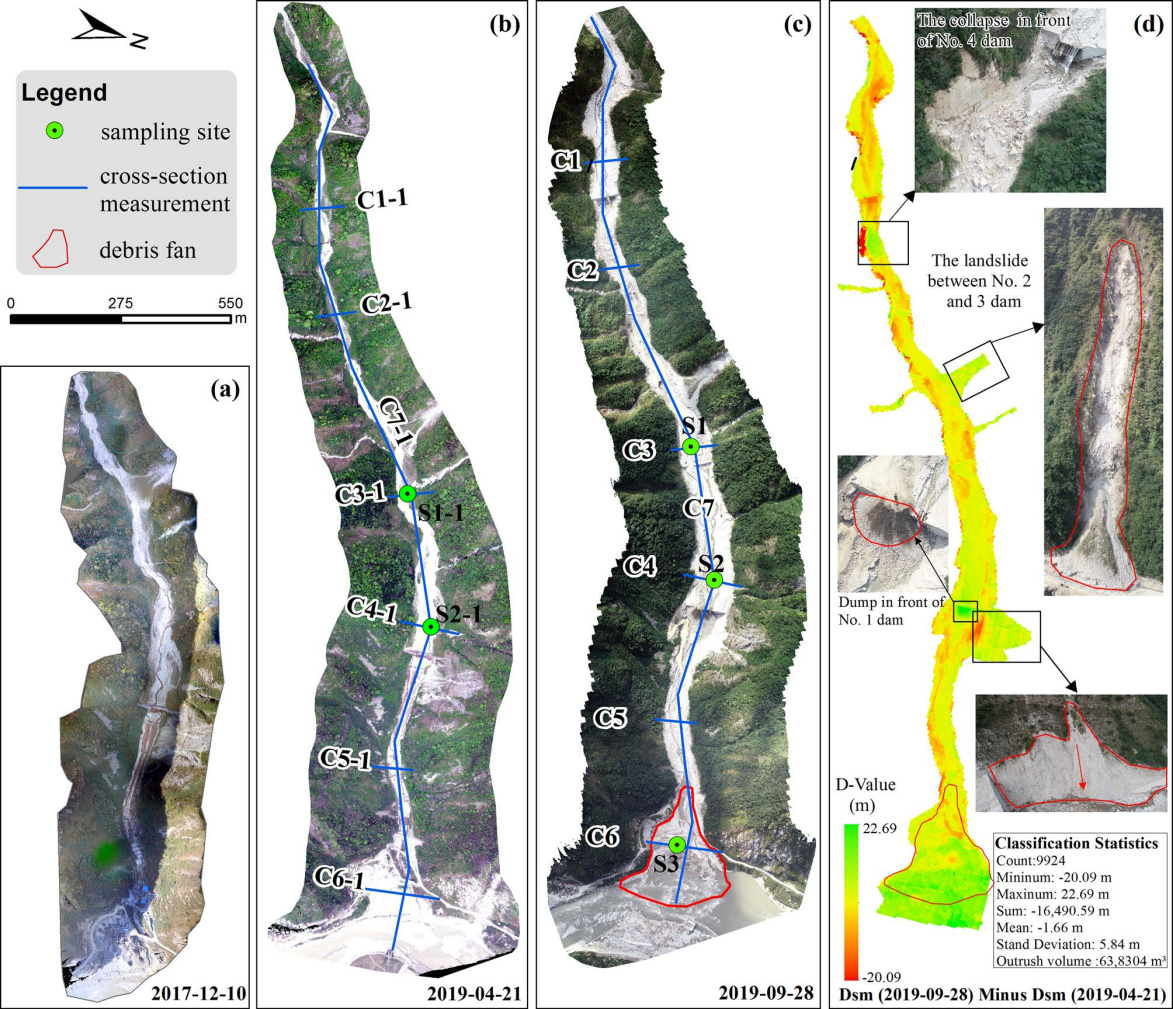
Field investigation sketch map. (**a**) Drone image taken on Dec. 10, 2017; (**b**) Drone image taken on Apr. 21, 2019, the distribution of sampling sites and cross (longitudinal)-section map are also noted; (**c**) Drone image taken on Sep. 28, 2019, the distribution of sampling sites and cross (longitudinal)-section map are also noted; (**d**) Raster image obtained by subtracting the DSM of April 21, 2019 from the DSM of September 28, 2019.Maps constructed using ArcGIS 10.2 (https://desktop.arcgis.com/en/).

Through interviews with local residents to understand the Chediguan gully debris flow history, as well as debris flow characteristics and other basic information. A 1:200 topographic map of the deposition area and a 1:200 cross (longitudinal)-section map of the downstream channel (C1-C6 and C1-1-C6-1 in Fig. 4) were created by UAV mapping method. As shown in Fig. 4, debris flow samples were collected at three sites (S1–S3) from the downstream channel and debris fan for sieving and particle gradation analysis (Fig. 4c). Field and laboratory dry sieving tests were conducted following the British Standards. Mostly composed of gravel and cobblestones. At the same time, field screening tests were carried out on the site of exposed sand and gravel. After recycling, weighing and drying, the materials were divided into 2–5, 5–10, 10–20, 20–60, 60–100, 100–150, 150–200 and greater than 200 mm. Fine materials (less than 2 mm in diameter) were further tested using laboratory dry screen tests.

### Drone aerial photography and measurement

We used a drone to photograph and map the areas below the No. 4 dam at Chediguan gully on December 10, 2017, April 21, 2019, and September 28, 2019, as shown in Table 2 and Fig. 4a–c. The accuracy of the terrain data obtained by the drone is as high as 1 cm, and the data can be used to accurately analyse the distribution of the source material and create a digital surface model (DSM). The DSMs obtained by the drone representing three periods were used to analyse the movement characteristics of sediments in the middle and lower reaches of the channel. Then, the differences in the DSMs between September 28 and April 21, 2019 was obtained by using the spatial analysis function of ArcGIS; these differences can reflect the topographic changes that occurred before and after the "8.20" debris flow (Fig. 4d).

**Table 2:** Data used in this study.

Data type	Source	Acquisition date	Resolution	Application
Images	Google Earth Images	2008/9/9	0.5 m	Generating provenance inventories
		2011/4/26		
		2015/4/15		
	Thematic Mapper	2019/8/7	5 m	
		2019/8/24		
	Drone aerial photography	2019/4/21	1 cm	Topographic survey
		2019/9/28		

The data showed that the "8.20" debris flow was mainly eroded from the middle and lower reaches of the channel, with an average erosion depth of 1.66 m, a total erosion depth of 1.64 × 10^4^ m^3^ and a total erosion amount of 39.59 × 10^4^ m^3^ (Fig. 4d). A large number of large-scale landslides and tributary debris fans on both sides of the main channel provided abundant loose material replenishment for the “8.20” debris flow, increasing the scouring force of the debris flow; thus, the drainage canal located before the No. 1 dam was completely destroyed.

Debris flows were mainly deposited in the gully mouth of the gully, and a total volume of 63.83 × 10^4^ m^3^ was deposited compared with the terrain surveyed on April 21, 2019; this number represents the accumulation amount of the "8.20" debris flow. As a large number of deposits congested the river, the riverbed of the Minjiang River was uplifted, and the river surface increased by 3.5 m on average before the outbreak.

### Multi-temporal source materials inventories

**Figure 5:**
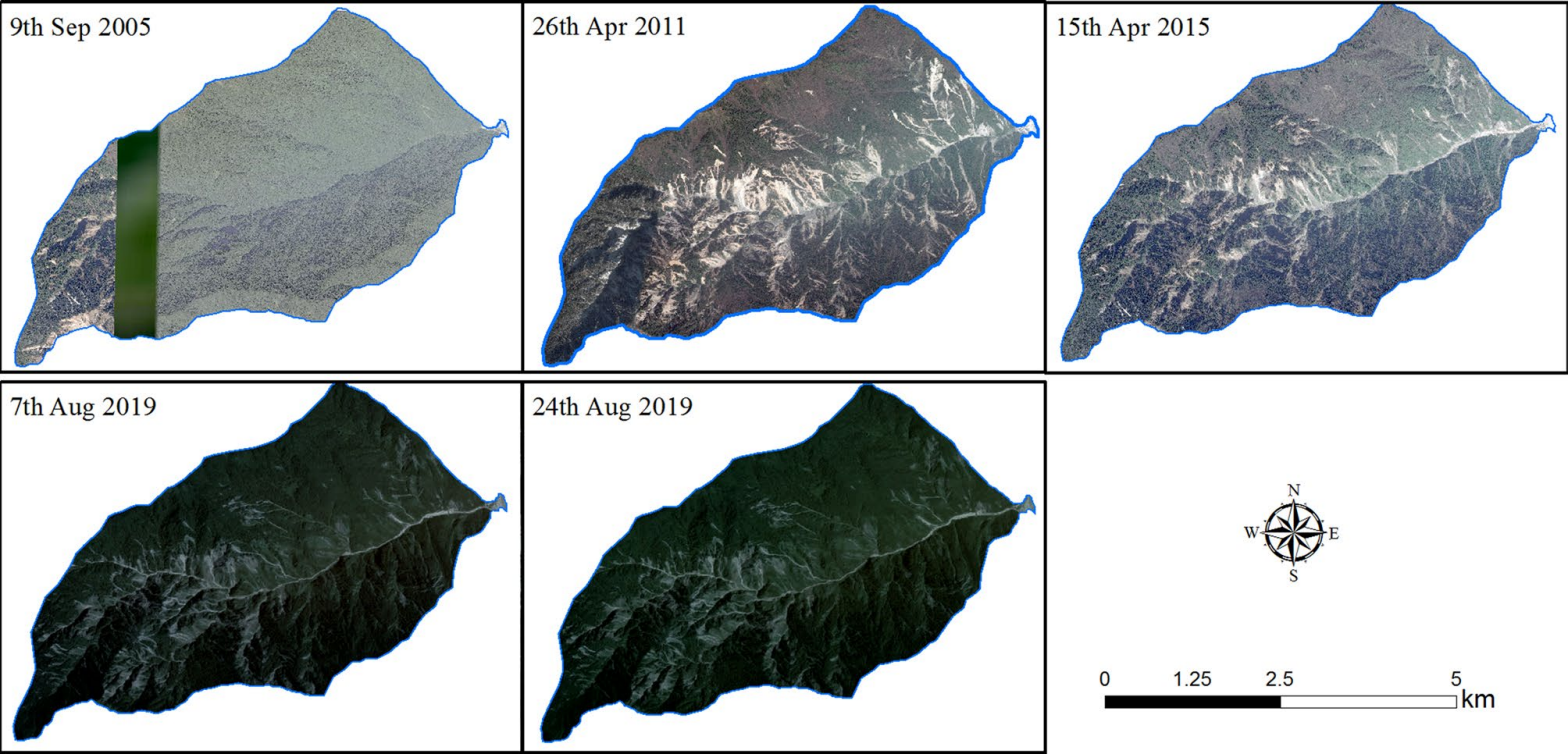
Digital stereoscopic images used in this paper.The data sources in the figure are shown in Table 2. Maps constructed using ArcGIS 10.2 (https://desktop.arcgis.com/en/).

Determining the provenance is the basic condition for studies of debris flows. Therefore, the key to determining the cause of a debris flow is to thoroughly determine the evolution law and migration form of the provenance of the associated debris. Based on this concern, a digital stereoscopic image interpretation was used to map the source material inventories (Table 2, Fig. 5). A resolution of 0.8 m on September 9, 2005, was used to reflect the source material conditions before the earthquake. Two Thematic Mapper (TM) satellite images with 30-m resolutions from the website of the US Geological Survey were used to analyse and compare the formation conditions of debris flows before and after the debris flow broke on 20th Aug 2019.

**Figure 6:**
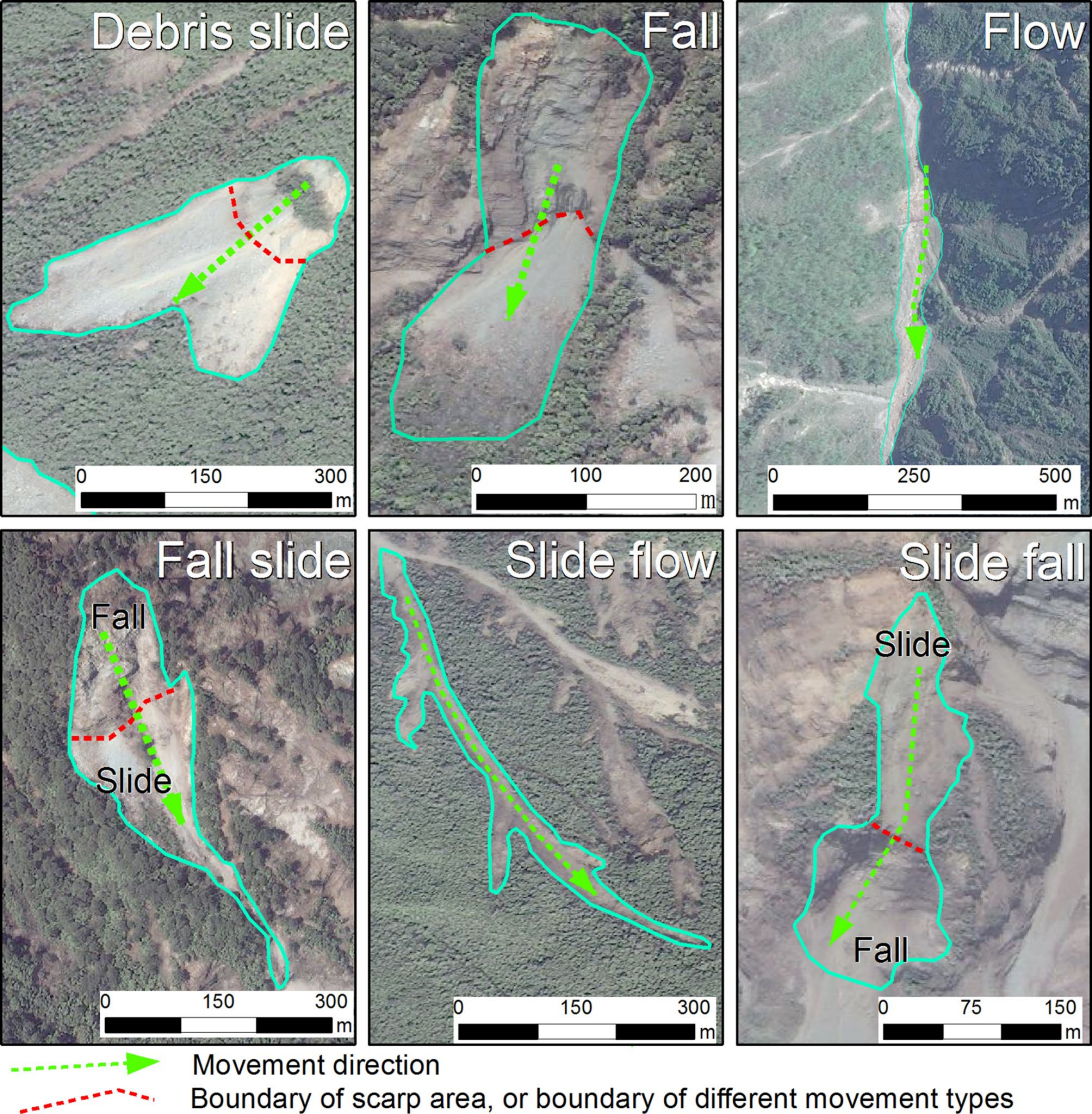
Examples of source materials movement types.Maps constructed using ArcGIS 10.2 (https://desktop.arcgis.com/en/).

The source materials were classified by their mass movement types^13^ (Fig. 6). We differentiated the following mass movement types: fall, slide, flow, fall-slide, slide-flow, and slide-fall. In the case of fall, materials fall from steep cliffs, with little additional displacement. Bedrock can be seen very clearly in the scarp area, and the accumulation area often tends to be cone-shaped. Slide-type movements are characterized by clear back scarps and the identification of a sliding mass that is either transitional or rotational in form. Flow-type movements are mostly confined to channels and occur mostly as debris flows. Fall-slide, a combination of falling and sliding, can be observed when fall-type movement occurs on a steep slope and the deposits slide down further during or after deposition. Slide-fall movements initiate as a slide on top of a steep cliff, and the slide materials subsequently fall over the cliff. A very common combination of landslide types is the slide-flow type, wherein the source material areas of a debris flow are formed by one or more slide-type movements.

### Parameter calculating

The unit weight is one of the most important parameters of a debris flow. It not only represents the concentration of the debris flow but also the necessary data for calculating the dynamic parameters of the debris flow. There are many methods for measuring the unit weight of a debris flow, among which the most accurate method involves field sampling and measurements. In addition, formulation methods and statistical formulas are also commonly used^14^. Due to the great subjective influence of witnesses, the accuracy of assessments of the grouting method cannot be guaranteed. Moreover, no suitable witnesses were found in the study area. Therefore, this paper chooses to use the statistical formula method to calculate the unit weight of the “8.20” debris flow^14^*.*

This method, through the statistical analysis of the debris flow using three characteristic particle sizes, represents the coarse particle size, the particle size of fine particles and the particle size of clay particles (2 mm, 0.05 mm and 0.005 mm, respectively) as well as the percentages of their relationships with the total debris flow unit weight and the correlations between the percentages of coarse and fine particles greater than 2 mm and less than 0.05 mm and the bulk density of the debris flow, as shown in formula (1)^14^.

1 $$\gamma_{D} = P_{05}^{ 0.35} P_{2} \gamma_{v} + \gamma_{0}$$

where P_05_ is the percentage of fine particles less than 0.05 mm (in decimal); P_2_ is the percentage of coarse particles larger than 2 mm (in decimal); γ_v_ is the minimum unit weight of viscous debris flow, = 2.0 g/cm^3^; γ_0_ is the minimum unit weight of the debris flow, = 1.5 g/cm^3^.

**Figure 7:**
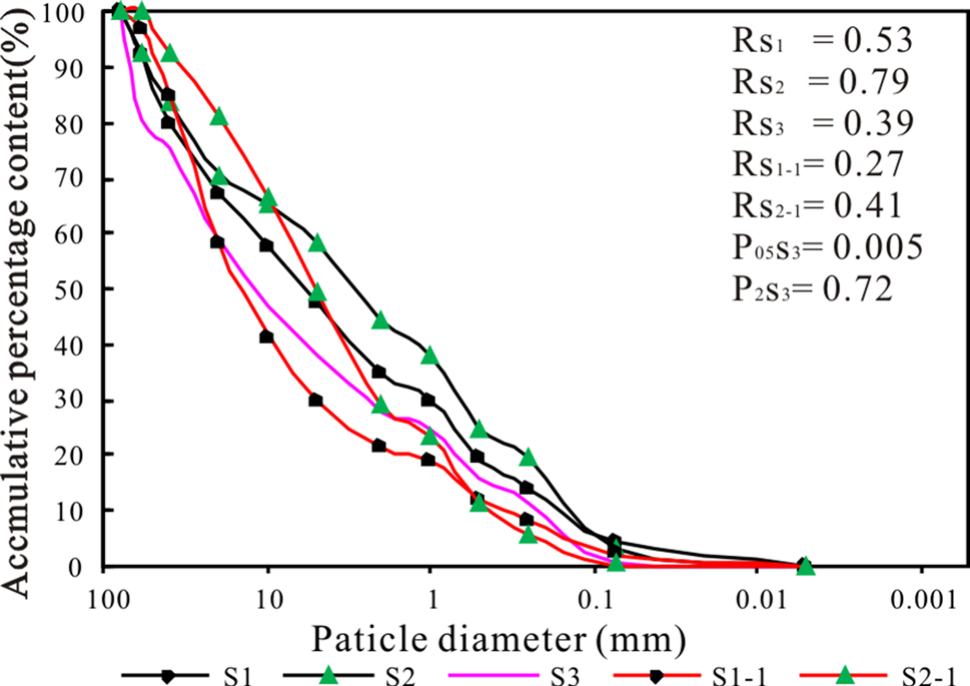
Particle size distributions of debris flow samples.

A summary of the parameters of the “8.20” Chediguan debris flow is shown in Table 3. The particle size distributions of debris flows are shown in Fig. 7.

**Table 3:** Parameters summary of debris flow samples.

Time	No	Site	P05	P2	R	γ_D_/g/cm^3^	Difference with 'S#-1'(%)			
							P_05_	P_2_	R	γ_D_
2019/9/28	S1	behind No.2 dam	0.044	0.654	0.53	1.938	50.00	− 20.03	48.23	1.34
	S2	behind No.1 dam	0.031	0.559	0.79	1.831	90.32	− 26.83	47.97	8.65
	S3	debris fan	0.005	0.72	0.39	1.725	–	–	–	–
2019/4/21	S1-1	behind No.2 dam	0.022	0.785	0.27	1.913	–	–	–	–
	S2-1	behind No.1 dam	0.003	0.709	0.41	1.686	–	–	–	–

### Calculation of dynamic process

The commonly used hydrological calculation formula is used to calculate the flood peak discharge and dynamic process of the debris flow. The formula is as follows.

#### Flood peak discharge

**Figure 8:**
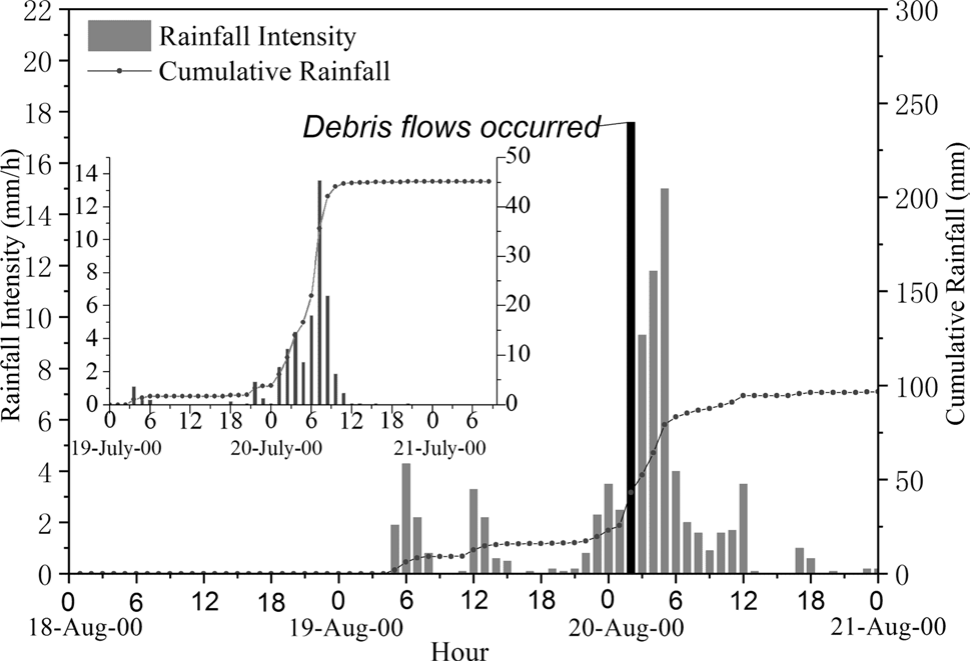
Distribution of hourly and accumulated rainfall on August 18–21, 2019.

Firstly, the flood peak discharge (Q_P_) of “8.20” Chediguan debris flow should be determined for the following calculation of debris flow peak discharge (Q_c_). The flood peak discharge (Q_P_) can be calculated by (2)^15^:

2 $$Q_{p} = 0.278\psi iF = 0.278\psi \frac{s}{{\tau^{n} }}F$$

where *F* is the catchment area; *ψ* is the runoff coefficient of flood peak; *S* is the maximum rainfall in an hour and equal to 17.8 mm/h; *τ* is the runoff confluence time of the rainstorm; and *n* is the attenuation index of the rainstorm. Figure 8 showed the rainfall distribution of hourly and accumulated rainfall on August 18–21, 2019. *ψ*, τ, and n can be determined by the following empirical equations^16^.

3 $$\psi = 1 - \frac{\mu }{S}\tau^{n}$$

4 $$\mu = (1 - n)n^{{\frac{n}{1 - n}}} (\frac{S}{{h^{n} }})^{{\frac{n}{1 - n}}} = 3.6F^{ - 0.19} \;(only\;applicable\; to \;Sichuan\;Province)$$

5 $$\tau = \left[ {\frac{0.383}{{mS^{1/4} /\theta }}} \right]^{{\frac{4}{4 - n}}}$$

6 $$m = 0.318\theta^{0.204}$$

7 $$\theta = \frac{L}{{J^{1/3} F^{1/4} }}$$

8 $$n = 1 + 1.285\left( {\log \frac{{H_{6p} }}{{H_{24p} }}} \right)$$

where *m* is the runoff confluence parameter; *θ* is the catchment characteristic parameter; *L* is the channel length; *J* is the longitudinal slope of the channel; *μ* is the runoff yield parameter; *H*_*6p*_ is maximum rainfall in 6 h and equal to 60.2 mm/h; *H*_*24p*_ is maximum rainfall in 24 h and equal to 60.2 mm/h.

#### Debris flow dynamic parameters

**Figure 9:**
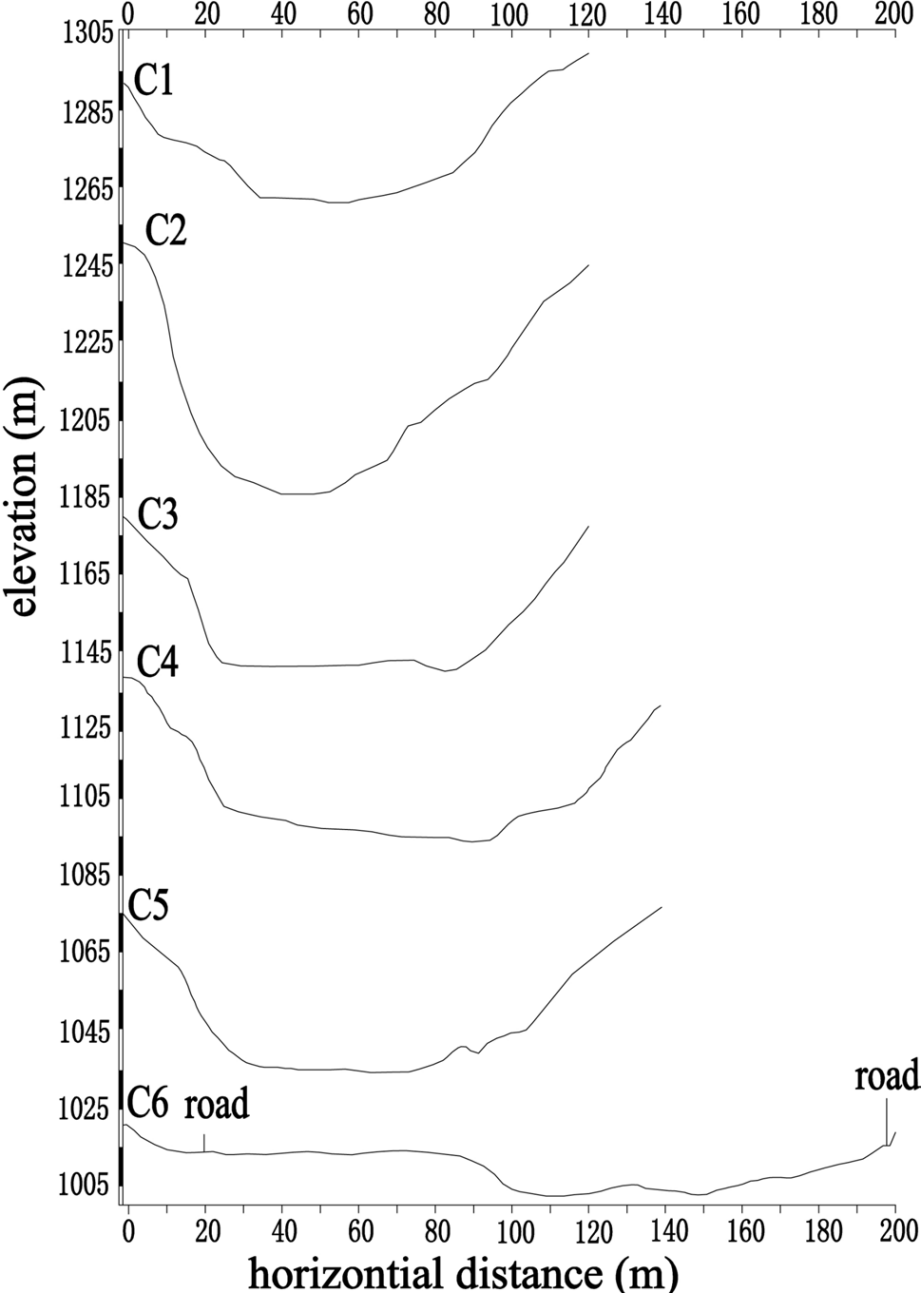
The measured cross-section (C1–C7).

For the following discussion on dynamic properties and hazard predictions, some dynamic parameters, including the debris flow velocity (*V*_*c*_), peak discharge (*Q*_*c*_), the total volume of one debris flow (*Q*_*t*_) and the debris flow impact force (*F*_*C*_), need to be determined. A total of 6 sections were selected for which to calculate these parameters. The distribution of these sections is shown in Fig. 1, and their terrain profiles are shown in Fig. 9.

The debris flow velocity (*V*_*c*_) can be calculated by^17^:

9 $$V_{C} = \frac{1}{{n_{C} }}\left( {H_{{_{C} }}^{2/3} \times I_{C}^{1/2} } \right)$$

where *H*_*c*_ is the hydraulic radius of the debris flow, can be replaced by the average deep mud; *I*_*c*_ is the hydraulic slope of debris flow (*J*), can be replaced by the longitudinal grade of channel; and *n*_*c*_ is the roughness coefficient and determined from an assignment table which is based on the debris flow fluid characteristic and channel condition^16^.

The debris flow peak discharge (*Q*_*c*_) can be calculated by two methods^16^:

10 $${\text{Stormwater}}\;\;{\text{method}}:Q_{c} = \left( {1 + \phi } \right)Q_{p} \cdot D_{c}$$

11 $${\text{ Morphological survey method}}:{\text{Q}}_{{\text{c}}} { = }W_{C} *V_{c}$$

where *D*_*c*_ is the debris flow blockage coefficient. Generally, the *Dc* is divided into three intervals according to the blockage degree: 1.0–2.5 (minor), 2.5–3.5 (normal), 3.5–4.5 (serious) and 4.5–5.5 (very serious). Based on the field investigation, the *D*_*c*_ of C1-C6 cross-section of Chediguan Gully is considered as 1.2–2.5 (Table3). *Ф* is the sediment correction factor of debris flow, which can be calculated by:

12 $$\Phi = \frac{{(\gamma_{c} - \gamma_{m} )}}{{(\gamma_{s} - \gamma_{c} )}}$$

where *γ*_*m*_ is the density of water (t/m^3^) (1.00 t/m^3^); *γ*_*s*_ is the density of the solid material (t/m^3^) and usually determined as 2.65 t/m^3^; and *γ*_*c*_ is the density of debris flow (t/m^3^). The *γ*_*c*_ listed in Table 2 was used for the calculation. As the soil samples are not enough to cover all the 6 sections, the *γ*_*c*_ value at each section is selected according to the nearby sample (Table 3). W_c_ is the area of debris flow cross section, which can be calculated by the mud depth and terrain lines (Fig. 9 and Table 3).

The total volume of one debris flow (*Q*_*t*_) can be calculated by^18^:

13 $${\text{Q}}_{{\text{c}}} = 0.0188{\text{Q}}_{{\text{t}}}^{0.79}$$

The impact force of debris flow is the direct force that causes damage to prevention engineering and buildings. The "8.20" debris flow had destroyed the no. 1 and 2 dam, the drainage channel in front of the no.1 dam, and two bridges in the gully mouth. Therefore, it is necessary to get the debris flow impact force (*F*_*C*_), which can help us understand the cause of these damages. The *F*_*C*_ can be calculated by^18^:

14 $${\text{F}}_{C} = \lambda \frac{{\gamma_{c} }}{g}V_{c}^{2} \sin \alpha$$

where λ is the form factor of building. Usually, λ is based on the shape of the building: circular (1.0), rectangular (1.33), square (1.47). α is the angle between the building surface and the direction of debris flow impact force (°). g is the gravitational acceleration (9.8 m/s^2^).

## Results and discussion

### Forming conditions

#### Triggering rainfall

Strong earthquakes cause the porosity of a source material to increase and become looser. At the same time, the source material becomes more prone to instability under the influence of rainfall and can then transforms into a debris flow. Therefore, the hydrodynamic conditions required for a debris flow are lower after an earthquake, as is the rainfall threshold^19^. Tang and Liang (2008) indicated that compared with the situation in Beichuan County before the earthquake, the critical hourly and cumulative rainfall decreased by 25.4–31.6% and 14.8–22.1%, respectively^20^.

According to the measured dataset at the rainfall monitoring station of Taoguan Village in Yinxing Township (Figs. 1, 8), rainfall began in the study area at 4:00 on August 19, 2019. The accumulated rainfall on that day was 23.1 mm. The accumulated rainfall before the debris flow broke out at 2 o'clock on August 20, 2019, was 20.1 mm; that is, the accumulated cumulative rainfall in the complete debris flow reached 43.2 mm.

The rainfall that eventually induced the debris flow appeared from 2:00 to 3:00 am on August 20, 2019. The maximum rainfall rate was only 17.8 mm/h, representing a slow-rising rain type. It should be noted that 48 h of rainfall also broke out in the area a month earlier, with a total rainfall of 45.2 mm and a maximum rainfall rate of 13.6 mm/h, which translated sediment from the tributaries and slope to the main channel and contributed to the debris flow on August 20, 2019. The typical debris flow events that have occurred in the past in Chediguan Gully are shown in Table 1. The accumulated rainfall amounts in the early stage of the mudslides that occurred on August 20, 2010, November 4, 2011, and November 20, 2011, were 55.3 mm (72 h), 102.8 mm (72 h), and 300 mm (72 h), and the excitation rain intensities were 19.5 mm/h, 14.2 mm/h, and 40 mm/h, respectively. Compared with past typical events, the pre-accumulated rainfall amount before the “8.20” debris flow was smaller than those in previous years, and the critical rainfall intensity decreased by 8.7% ~ 55.5%.

#### Sediment supply conditions

**Figure 10:**
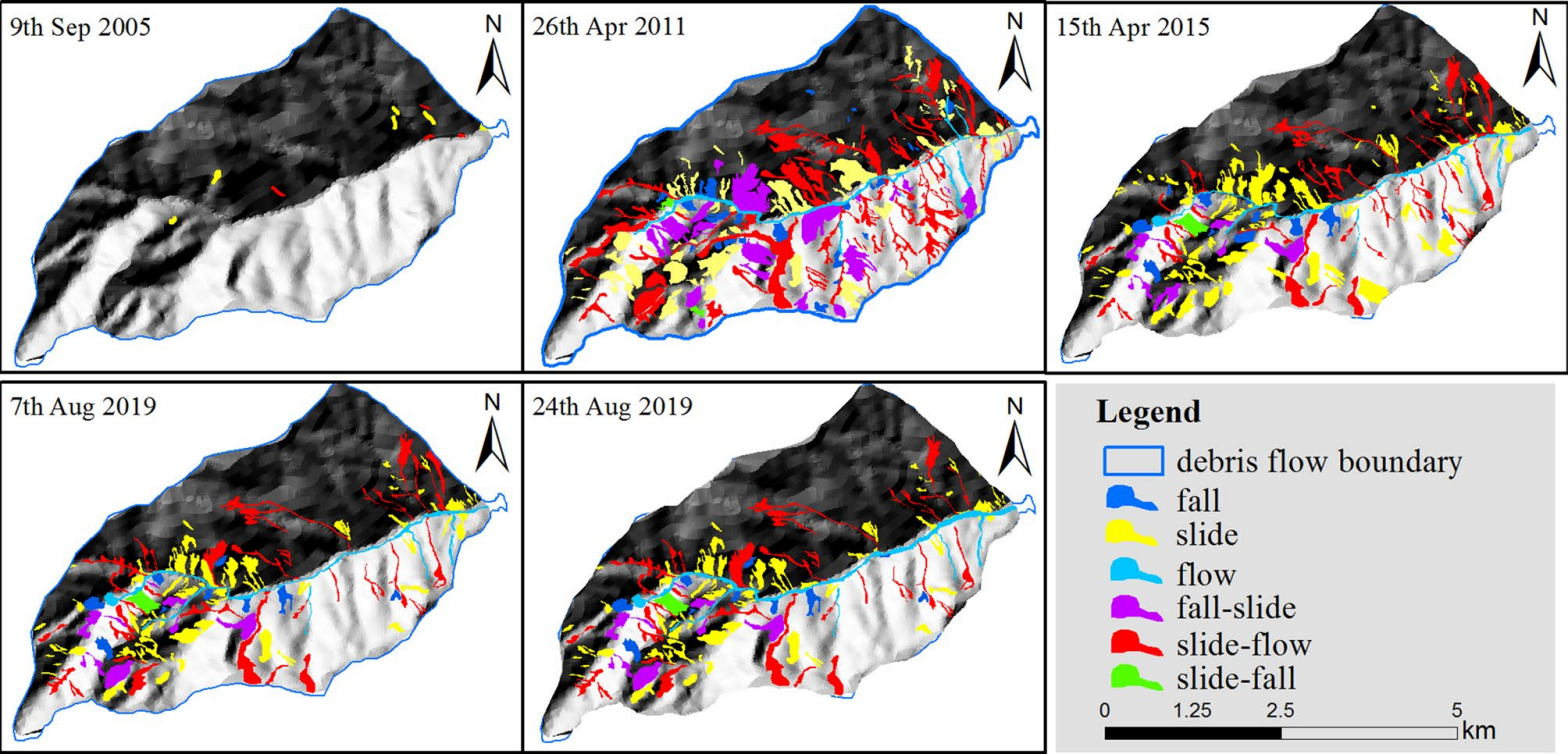
Multiphase provenances distribution map of Chediguan Gully. Maps constructed using ArcGIS 10.2 (https://desktop.arcgis.com/en/).

The multiphase source distribution of Chedaiguan gully is shown in Fig. 10. The changes in source materials that occur with different movement types are shown in Table 4 and Fig. 10. As shown in Table 4, before the earthquake, the total area of source materials developed in Chediguan gully was only 2.86 × 10^4^ m^2^, which increased to 473.8 × 10^4^ m^2^ in 2011. After the earthquake, from 2011 until August 2018, the number of source materials in the study area decreased by 83 to a total of 123. The total source material area increased from 473.8 × 10^4^ m^2^ to 265.6 × 10^4^ m^2^. The total amount of source materials in Chediguan gully after the “5.12” earthquake shows a continuously decreasing trend, while source materials that underwent different movement types show different change rates.

**Table 4:** Statistics of the source materials inventories before and after the earthquake.

Time	Movement types												Total	
	Fall		Slide		Flow		Fall-slide		Slide-flow		Slide-fall			
	Number	Area	Number	Area	Number	Area	Number	Area	Number	Area	Number	Area	Number	Area (× 10^4^m^2^)
2005/9/9	0	0	6	1.83	0	0	0	0	4	1.03	0	0	10	2.86
2011/4/26	27	27.5	83	122.5	7	28.2	25	95	62	197.8	2	2.8	206	473.8
2015/4/15	11	24.1	90	129.9	5	23.7	9	24.9	41	91.1	1	6.4	157	300.1
2019/8/7	9	17.8	63	89.9	5	26.4	9	26.7	36	99.3	1	5.5	123	265.6
2019/8/24	10	18.2	67	95.2	5	34.8	9	26.7	35	96.4	1	6.4	127	277.7

**Figure 11:**
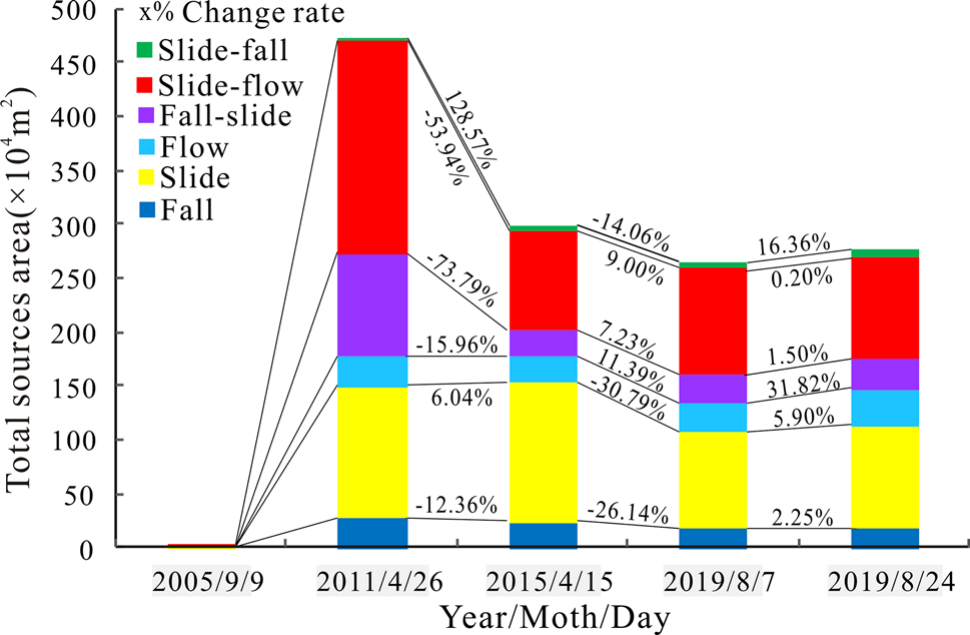
Change of source materials with different movement types based on multiple phases of remote sensing images.

The results show (Fig. 11) that the total area of the flow-moving source materials increased by 6.04% from 2011 to 2015, while the other areas decreased considerably. Between 2015 and August 7, 2019, the total areas of source materials moving as slide-flow, fall-slide, and flow types increased by 9%, 7.23% and 11.39%, respectively, while the total source material areas moving as slide-fall, slide, and fall types decreased by 14.06%, 30.79% and 26.14%, respectively. After the debris flow occurred on August 20, 2019, the number of source materials in Chediguan gully increased to 127. The total source material area increased to 281.2 × 10^4^ m^2^. Among the source materials, the areas of source materials moving as slide-falls and flows increased the most, with growth rates of 16.36% and 31.82%, respectively; these were also the main source material of the "8.20" debris flow sediment supply.

The observed changes in the data indicate that the source materials after the “5.12” earthquake evolved in different ways. First, the slide-flow and slide types are the main movement types of the provenance material. In 2011, the source materials moving in these two movement types accounted for 41.75% and 20.05% of the total, respectively, while the data on August 24, 2019 showed 35.38% and 33.85% of the total. Second, the source materials were continuously moved to the lower region in the form of slide-flows, fall-slides, and flows under the effects of runoff erosion and gravity. The third finding is that the source material in the watershed mostly moves as the flow movement type, and its area is expanded by the formation of lateral and downward erosion (Fig. 4); this erosion was the main cause of the "8.20" debris flow. At present, the provenance material in Chediguan gully is mainly distributed in the main gully and moves in the form of slide-flows and flows. Therefore, dredging work in the main channel is necessary to reduce the possibility of debris flows.

#### Deposition characteristics

**Figure 12:**
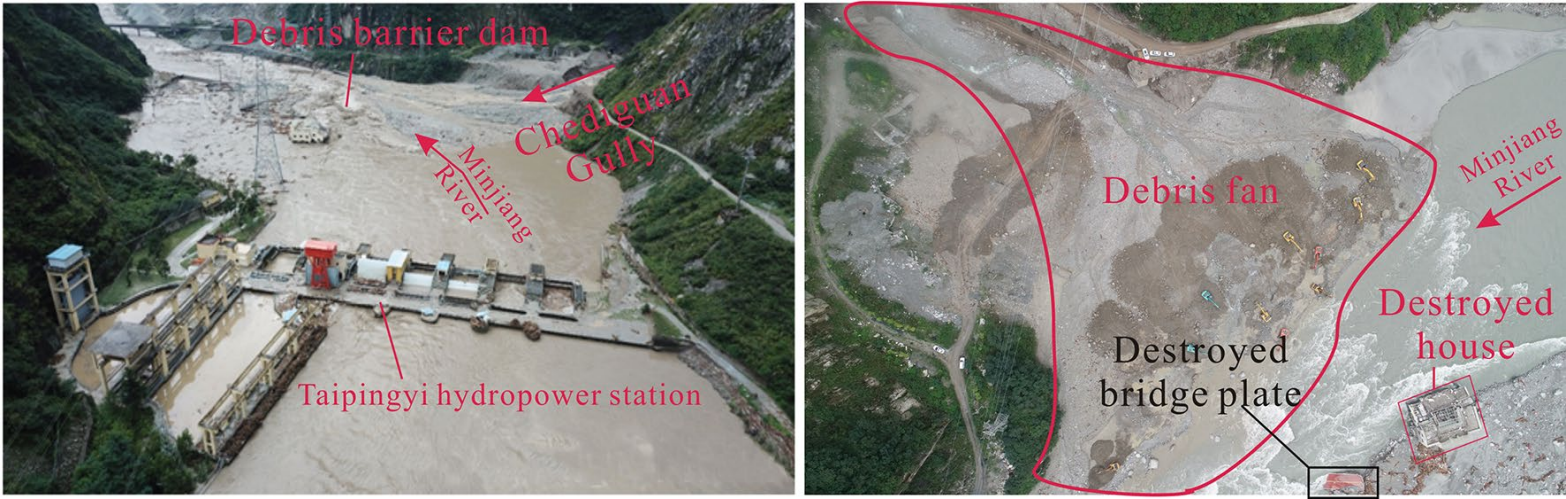
Debris fan and barrier lake formed in the “8.20” debris flow event.

**Figure 13:**
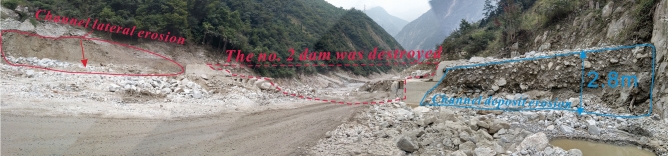
Longitudinal cross-section of the “8.20” debris flow event.

In the '8.20' event, debris flow material was transported to the gully mouth and formed a large debris flow fan. (Fig. 12). The fan was 300 m long and 260 m wide, with an average depth of 8 m (Fig. 4). The aerial photos show that the debris flow fan area was 3.1 × 10^4^ m^2^ and the volume was about 63.8 × 10^4^ m^3^. The debris flow destroyed the bridge at the gully mouth, destroyed many houses at the opposite bank of the Mingjiang River, and finally silting into a debris fan. The Fig. 13 shows the significant changes that occurred in the middle-lower gullies and gully mouth before and after the “8.20” debris flow event. The debris flow consists mainly of erosion before the C6 cross-section and mainly of deposition after the C6 cross-section. The highest deposition depth at the gully mouth reached 9.5 m, the riverbed was uplifted, and nearly 1/3 of the river was buried. The debris flow blocked the Minjiang River temporarily during the flood peak, causing the river to return and flood the Taipingyi hydropower station 200 m upstream, leaving two plant workers missing.

To obtain the particle grading of the debris flow granules, we went to Chedgiuan gully on April 21, 2019, and September 28, 2019, and obtained a total of 5 soil samples (S1, S2, S3, S1-1, and S2-1). The sampling positions are shown in Fig. 4c. The sampling point S3 was located in the middle of the debris fan and can be used to calculate the debris flow unit weight. The particle size distributions of the three sets of soil samples obtained by sieving are shown in Fig. 5. The P05 of S3 is 0.005, and its P2 is 0.72. By substituting these values into formula (1), it can be obtained that the unit weight of the debris flow was 1.725 g/cm, which is larger and belongs to the class of sub-viscous debris flows. According to the survey conducted by the Sichuan Metallurgical Geological Exploration Bureau, the unit weight of the debris flow that occurred on July 20, 2011, was 1.866 t/m^3^, higher than the unit weight of the "8.20" debris flow.

Particle gradations at different locations and over different periods can visually reflect the transport processes of different particles during debris flows. The data of all the samples are shown in Table 2 and Fig. 7. It can be seen that the P05 of the soil sample collected in the channel after dam No. 1 increased by 50%, the P2 decreased by 20.03%, and the earth-rock ratio increased by 48.23%, while the unit weight increased by 1.34%. The soil samples collected after dam No. 2 showed similar trends, with the P05 increasing by 90.32%, the P2 decreasing by 26.83%, the earth-rock ratio increasing by 47.97%, and the unit weight increasing by 8.65%. These changes in the collected data show that after the debris flow events, the fine particle content measured after the dam and the soil-rock ratio were greatly increased, while the coarse particle content was reduced. The parameter differences among S1, S2, and S3 reflect the particle movement process during the "8.20" debris flow. The P05 values of S1, S2, and S3 were 0.044, 0.031, and 0.005, and the P2 values of S1, S2, and S3 were 0.654, 0.559, and 0.72, respectively. In other words, the fine particle content gradually decreases from the upstream region to the debris fan, while the coarse particle content increases continuously.

Therefore, the "8.20" debris flow was mainly formed by coarse particles, and a large number of fine particles from upstream were blocked by the dams. The interception of the dam caused an increase in the soil-rock ratio and soil unit weight of the sediment behind the dam. Coarse particles continued to move downstream with the debris flow, thereby reducing the unit weight of the debris flow sediment.

### Formation mechanism and dynamic characteristic

#### Formation mechanism

The previous analysis shows that the “8.20” Chediguan debris flow event had the following characteristics: (1) the rainfall that occurred one month before the debris flow brought the sediment to the main channel together; (2) the source material was started collectively by downcutting of the broached groove; (3) the debris flow movement was mainly undercut erosion and lateral erosion in the main channel; and (4) the impact force of the debris flow was extremely high.

As shown in Fig. 12, the total areas of the provenance materials experiencing slide-flow, fall-slide, and flow movement on August 7, 2019 were 9%, 7.23%, and 11.39% higher than that of 2015, respectively, and the bright colors shows that these movements occurred recently. Figure 8 shows that the concentrated rainfall that occurred on July 18–20, 2019, is one of the causes of sediment movement. Heavy rainfall caused the initiation of some landslides and the initiation of debris flows in some tributaries.

**Figure 14:**
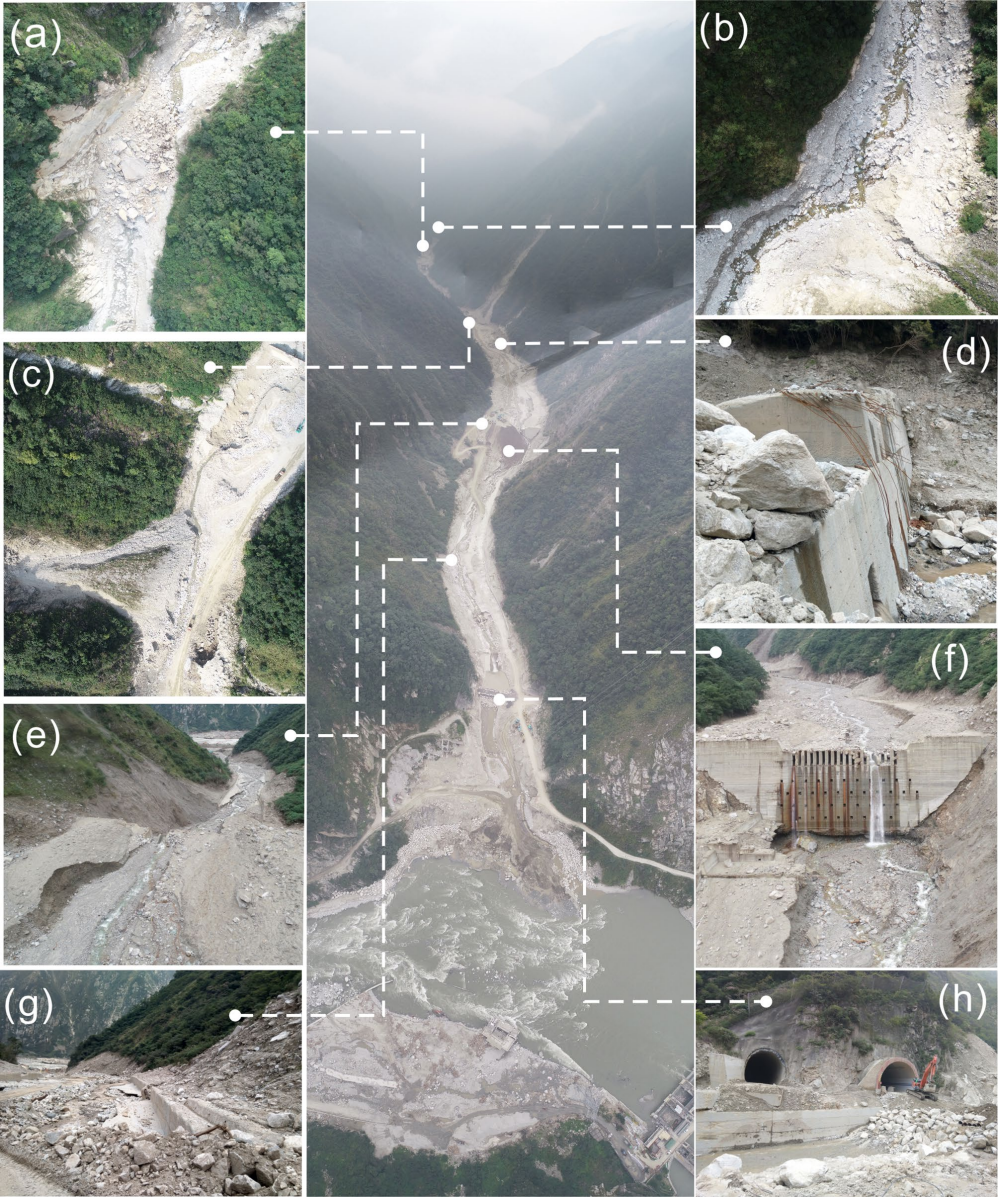
Characteristics maps of Chediguan debris flow broken in 20th August. (**a**) the collapse in front of no. 4 dam due to erosion by water flow; (**b**) the debris fan of no.8 tributary was eroded; (**c**) the old deposition fan and slope behind no 2 dam was eroded; (**d**) the no. 2 dam was destroyed; (**e**) channel erosion characteristics below no 1 dam; (**f**) the channel in front of no. 1 dam was severely down-eroded; (**g**) the drain groove at the gully mouth was completely destroyed; (**h**) two Bridges at the gully mouth were washed away. Maps constructed using Coreldraw × 7 (https://www.corel.com/cn/).

During the downward migration of the tributary debris flows, the coastal sediments were continuously washed and finally entered the main channel. Within one month before the “8.20” debris flow, the sediments continued to move toward the main channel, providing abundant provenance conditions for the debris flow. The previous rainfall also gave the old deposits a higher moisture content, making them easier to initiate into a debris flow. According to the conducted interview, the Chediguan debris flow began at approximately 2 a.m. on August 20th, 2019. Heavy rainfall again induced mudslides in the tributary. The rushing material of the upstream tributary entered the main channel to form a debris barrier dam at a great rate, eroded by the upstream inflow by cutting down the groove (Fig. 14a–c,e), and finally converted to a mountainous torrent and debris flow.

**Figure 15:**
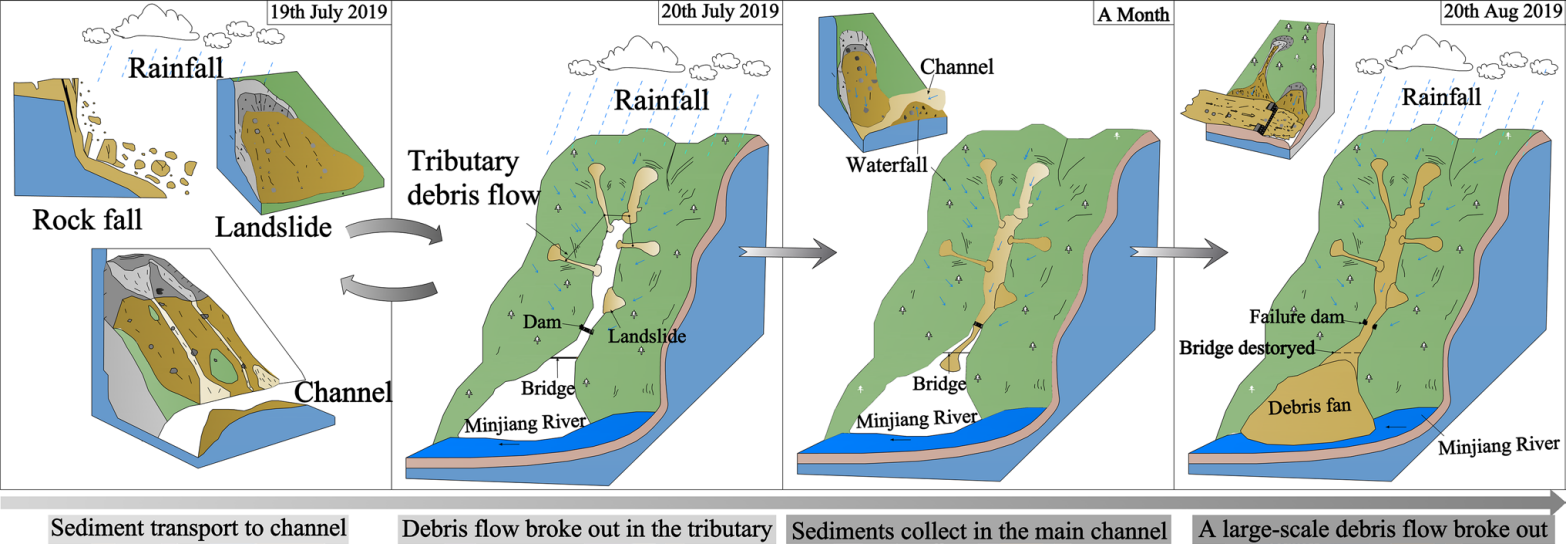
Formation process sketch maps of Chediguan debris flow broken in 20th August. Maps constructed using Coreldraw × 7 (https://www.corel.com/cn/).

The upstream debris flow rapidly expanded downstream via forward erosion an continuously revealed sediments along the way (Fig. 14 a–c,e). The debris was like a “snowball” that became increasingly larger, destroying three sand-blocking dams and bridges in the middle and lower reaches of the channel (Fig. 14d,f–h). Finally, the debris flow rushed into the Minjiang River, caused a short-term blockage, and then deposited and formed a debris fan in the gully mouth section. In conclusion, the Chediguan debris flow had an obvious chain effect, characterized by the combination of heavy rain (mountainous torrent)-collapse landslide-tributary debris flow-rainfall-channel erosive erosion to form the debris flow. The formation process of the “8.20” Chediguan debris flow is shown in Fig. 15.

#### Dynamic characteristic

According to the formula listed in Sect. 2.5 above, the dynamic parameters of the debris flow in different channel sections were calculated and are listed in Table 5. It is worth noting that the amount of debris flow calculated by the morphological survey method is closer to the measured value of 63.8 × 10^4^ m^3^ than the value obtained by the rain flood method. Because the rainfall data used in the rain flood method were measured from the position of the gully mouth, the actual rainfall in the formation region upstream of the ditch may be far greater than the rainfall data we used. This result also confirms the reliability of the parameters such as the river flow rate, impact force and debris flow rate calculated by the morphological survey method.

**Table 5:** Calculation results of related parameters.

Calculation content	Units	Section number						
		C1	C2	C3	C4	C5	C6	
H_c_	(m)	2.8	2.4	3.5	1.7	1.9	7.5	
B_c_	(m)	46.5	45.8	76.3	81.3	67.9	106.2	
W_c_	(m^2^)	128.54	202.86	329.01	217.37	166.48	459.81	
J	(‰)	185.29	80.1	141.28	54.59	132.41	113.15	
γ_c_	(t/m^3^)	1.94	1.94	1.94	1.83	1.83	1.73	
F	(km^2^)	11.24	12.2	13.63	15.32	16.35	16.49	
ψ	/	2.54	2.62	2.79	2.87	2.99	3.12	
τ	h	2.79	2.90	3.09	3.19	3.32	3.49	
S	mm/h	17.8	17.8	17.8	17.8	17.8	17.8	
n_c_	/	0.27	0.07	0.10	0.08	0.03	0.05	
ϕ	/	1.32	1.32	1.32	1.01	1.01	0.79	
L	(km)	4.49	4.75	5.24	5.58	5.94	6.26	
D_c_	/	2.5	2.3	2	1.9	1.5	1.2	
λ	/	1.33	1.33	1.33	1.33	1.33	1.33	
α	°	90	90	90	90	90	90	
V_c_	(m/s)	3.17	5.49	9.35	2.64	5.49	11.23	
Stormwater method	Q_P_	(m^3^/s)	32.86	35.34	38.68	43.19	45.46	45.02
	Q_c_	(m^3^/s)	190.912	188.8948	179.7803	165.1227	137.2116	96.89087
	Q_t_	(m^3^)	51,775.33	51,228.27	48,756.41	44,781.29	37,211.78	26,276.8
Morphological survey method	Q_c_	(m^3^/s)	407.1118	1114.649	3075.526	574.3737	914.795	5161.909
	Q_t_	(m^3^)	55,204.35	151,146.4	417,041.3	77,885.07	124,046.2	699,954.8
	*F*_*C*_	(Pa)	26.40964	79.49356	230.0625	17.34015	74.9862	295.8936

## Discussion

Eleven years after the “5.12” Wenchuan earthquake, such a large-scale debris flow can still erupt in the earthquake zone, indicating that the region is still in the active period of earthquake geological disasters; the reasons behind this activity should be the focus of our work. In this paper, we investigated the debris flow disaster scene that occurred on August 20, 2019 and analyzed the formation mechanism and dynamic characteristics of this debris flow event by means of a drone survey, a high-definition remote sensing interpretation and other means. However, at the same time, through this research, we also found many problems that should be considered in future research; these issues mainly include the following two points.

1. The acquisition of rainfall data in debris flow formation areas may not be representative. As one of the conditions necessary for the formation of debris flows, rainfall is usually the most important inducing factor of debris flows^21^, so collecting rainfall data during debris flows should be the most important task in research; however, this is not an easy task. The current rainfall monitoring stations are limited and are mostly located in the lower gully mouth area. However, a large number of studies have shown that rainfall in debris flow basins increases with elevation, so rainfall in debris flow formation areas is often much higher than in ditch locations^22^. Our research results also prove this. Therefore, in future work, we should install more rain collection instruments in key debris flow gullies, especially in debris flow formation areas, and avoid using the rainfall data representing the ditch region to calculate dynamic parameters such as the flow rate of the river and the flow rate of the debris flow.
2. The provenance material amount may be underestimated. The current estimation of the provenance material amount in the debris flow basin mainly determines the area by means of optical interpretation and then estimates the volume using a statistical formula^17^*.* The problem with this method is that the estimation of the movable depth of the provenance is often unable to reflect the specific starting depth, which will lead us to underestimate the actual provenance. Through the research conducted in this paper, it was found that the area of earthquake-induced loose deposits decreases sharply over time, but this does not mean that the possible material provenance also decreases. The results of our study on the evolutionary characteristics of provenance materials in multiple stages with different movement types show that during the 11 years after the earthquake, the provenances continue to move through slide-flow and flow movements under the actions of runoff and gravity. These moving sediments eventually settle thicker and thicker in the gradual areas and wait to be restarted by rainfall. A more accurate provenance depth can only be determined by drilling or geophysical analysis, and the depth that can be initiated may need to be further determined by measuring the shear strength of the formation. In addition, the depth that can be initiated is also related to the actual volume of runoff. The different flow strengths must be able to initiate the provenance at different depths.
3. Why the scale and damage of the "8.20" debris flow were higher than ever under less rainfall. This phenomenon also shows that rainfall is not the only cause of debris flow events. Our findings show that there may be three reasons for this phenomenon. More source material in the main channel is the first and most important reason. The investigation found that after the debris flow broke out on July 20, 2011, the No. 1 to No. 4 dams were filled with sediments from the tributaries and slopes. The second reason is the rainfall that occurred on July 19–21, 2019. This rainfall event transported the source materials in the tributaries and slopes into the main channel and at the same time gave these materials a higher moisture content, making them easier to be initiated into a debris flow. The third reason is the difference in the rainfall data. Our rainfall data are taken from lower altitudes than that of the study site, while debris flows usually form at higher elevations. Studies show that higher rainfall occurs at higher elevations^22, 23^. Therefore, our rainfall data cannot represent the actual rainfall that triggered the "8.20" debris flow. We will continue to explore the possibilities for these reasons in the following chapters.

In this paper, the calculation of the morphological investigation method compensates for the error caused by the lack of rainfall data, but this calculation still cannot accurately indicate the current accurate provenance volume. Although we have accurate provenance areas, we cannot determine the depth of all provenances. In addition, because there are no field survey data or high-resolution images of the debris flow formation area, we cannot analyse the actual start-up process of the debris flow in detail; thus, we need to supplement a detailed investigation of the upstream channel and formation area in the next study.

## Summary

Eleven years after the Wenchuan earthquake, a large-scale debris flow could still break out in Chediguan gully in the earthquake zone, causing more serious losses than before. Studies of the cause and characteristics of the "8.20" debris flow are of great significance for further understanding the status and activity of the debris flow after the Wenchuan earthquake. Based on this concern, this paper investigated the Chediguan debris flow disaster scene that occurred on August 20, 2019 and analysed the formation mechanism and dynamic characteristics of this debris flow event by means of a drone survey, a high-definition remote sensing interpretation and other means.

The main conclusions of this paper are as follows:

1. The accumulated rainfall before the outbreak of the Chediguan debris flow at 2:00 on August 20, 2019, was 20.1 mm, and the accumulated rainfall in the previous period totalled 43.2 mm. The simulated rainfall that eventually induced the debris flow appeared on the morning of August 20, 2019, from 2:00 to 3:00 in the morning. The maximum rainfall intensity was only 17.8 mm, representing a slow-rising rain type.
2. After the earthquake, from 2011 until August 2018, the number of provenances in the study area decreased by 83 to a total of 123. The total provenance area increased from 473.8 × 10^4^ m^2^ to 265.6 × 10^4^ m^2^. The total amount of provenance in the research area after the “5.12” earthquake showed a continuously decreasing trend, but the provenances with different movement types showed different change rates. The total area of the flow-moving provenances increased by 6.04% from 2011–2015, while the others decreased considerably. Between 2015 and August 7, 2019, the total area of provenances moving as slide-flows, fall-slides, and flows increased by 9%, 7.23% and 11.39%, respectively, while the total areas of provenances moving as slide-falls, slides, and falls decreased by 14.06% and 30.79%, and 26.14%, respectively. After the debris flow broke on 20th Aug 2019, the number of provenances in the study area increased to 127. The total provenance area increased to 281.2 × 10^4^ m^2^. Among the flow types, the areas of provenances moving in slide-falls and flows increased the most, with growth rates of 16.36% and 31.82%, respectively; these were also the main source material of the "8.20" debris flow sediment supply. At present, the provenance in Chediguan gully is mainly distributed in the main gully and moves in the form of slide-flows and flows. Therefore, a large amount of dredging work in the main gully can effectively reduce the risk in the gully.
3. The previous analysis of this paper shows that the “8.20” Chediguan debris flow event has the following characteristics: (1) pre-rainfall brought the sediment together; (2) the flow cut down the groove, concentrating the initiation of the provenance; (3) the main activity involved lifting erosion in the main channel; and (4) the debris flow had an extremely high impact force. The Chediguan debris flow had an obvious chain effect, which is characterized by the combination of heavy rain (mountain torrent)-collapse landslide-tributary debris flow-rainfall-channel erosive erosion to form the debris flow. According to the parameter calculation, the "8.20" Chediguan debris flow was a sub-viscous debris flow, the unit weight of the debris flow was approximately 1.725 g/cm^3^, the maximum velocity at the gully mouth was 11.23 m/s, the peak flow was m/s^3^, the overall impact force of the debris flow at the gully mouth reached 295.9 Pa, and the total volume of the debris flow exceeded 70 × 10^4^ m^3^. The difference between the measured number of outflowing debris (63.8 × 10^4^ m^3^) and the calculated result was 1.55%, and the high coincidence between these values reflects the accuracy of the calculated results in this paper.

The study shows that the impacts of the Wenchuan earthquake on the debris flow in the earthquake zone still exist. The geological disasters in the Wenchuan earthquake zone are still in an active period, and earthquake zone is still at a high risk for debris flows. Taking into account the causes and characteristics of historic debris flows, detailed investigations and assessments of debris flows in the earthquake zone should be carried out to execute the necessary dredging work for the watershed with excessive accumulation behind the dam. In addition, it is important to carry out the necessary repair or rebuilding of damaged mitigation measures, restore and enhance resilience to minimize the damage caused by debris flows, and establish a real-time monitoring and early warning system for potentially high-risk debris flows to ensure early detection, early prevention, and early management.
